# Warming seas increase cold-stunning events for Kemp’s ridley sea turtles in the northwest Atlantic

**DOI:** 10.1371/journal.pone.0211503

**Published:** 2019-01-29

**Authors:** Lucas P. Griffin, Curtice R. Griffin, John T. Finn, Robert L. Prescott, Mark Faherty, Brett M. Still, Andy J. Danylchuk

**Affiliations:** 1 Department of Environmental Conservation, University of Massachusetts Amherst, Amherst, Massachusetts, United States of America; 2 Massachusetts Audubon Society, Wellfleet Bay Wildlife Sanctuary, South Wellfleet, Massachusetts, United States of America; 3 Department of Natural Resources Science, University of Rhode Island, Kingston, Rhode Island, United States of America; Florida State University, UNITED STATES

## Abstract

Since the 1970s, the magnitude of turtle cold-stun strandings have increased dramatically within the northwestern Atlantic. Here, we examine oceanic, atmospheric, and biological factors that may affect the increasing trend of cold-stunned Kemp’s ridleys in Cape Cod Bay, Massachusetts, United States of America. Using machine learning and Bayesian inference modeling techniques, we demonstrate higher cold-stunning years occur when the Gulf of Maine has warmer sea surface temperatures in late October through early November. Surprisingly, hatchling numbers in Mexico, a proxy for population abundance, was not identified as an important factor. Further, using our Bayesian count model and forecasted sea surface temperature projections, we predict more than 2,300 Kemp’s ridley turtles may cold-stun annually by 2031 as sea surface temperatures continue to increase within the Gulf of Maine. We suggest warmer sea surface temperatures may have modified the northerly distribution of Kemp’s ridleys and act as an ecological bridge between the Gulf Stream and nearshore waters. While cold-stunning may currently account for a minor proportion of juvenile mortality, we recommend continuing efforts to rehabilitate cold-stunned individuals to maintain population resiliency for this critically endangered species in the face of a changing climate and continuing anthropogenic threats.

## Introduction

Historically, sea turtle populations experienced wide-spread declines, primarily from by-catch and harvest of adults and eggs [1]. While conservation measures have helped to increase sea turtle populations globally [2], both fine- and large-scale threats persist for all seven species of sea turtles, including bycatch, harvest, habitat degradation, pollution, tourism, and climate change. Of these, climate change may present the broadest threat for sea turtle conservation [3–5]. Predicted warmer temperatures and sea level rise may decrease hatchling success and available nesting habitats, and skew sex ratios [5,6].

Less is understood about the potential effects of climate change on sea turtle cold-stunning events. As a result of prolonged exposure to cold water temperatures, hypothermic cold-stunned sea turtles can experience debilitating lethargic conditions that often lead to death [7–10]. All sea turtle species are susceptible to becoming cold-stunned; however, the Kemp’s ridley (*Lepidochelys kempii*), loggerhead (*Caretta carreta*), and green turtle (*Chelonia mydas*) are the most frequently cold-stunned species in the U.S., with cold-stun stranding events occurring at the upper limits of their ranges both in low and high latitudes [11]. At lower latitudes, cold-stunning events are acute and triggered by extreme cold weather snaps, often during relatively mild winters [12–15]. At higher latitudes, cold-stunning events are associated with turtles not migrating south before the onset of late autumn storms and associated declining seasonal water temperatures [16–20]. In both regions, cold-stunning events occur when turtles appear to be unexpectedly caught in areas with lower water temperatures and fail to depart from shallower colder nearshore waters. For example, in 2010, approximately 5,000 juvenile green turtles were cold-stunned and stranded across Florida [13], while in 2014, over 1,100 Kemp’s ridleys stranded in Cape Cod Bay, Massachusetts.

Dependent on local wind and oceanic currents, cold-stunned sea turtles typically wash-up on beaches where, if found prior to death, they are recovered and sent to rehabilitation centers [18]. These recovery programs can be highly effective at reducing mortality rates of cold-stunned turtles. For example, in the large-scale 2010 Florida cold-stun event, only 20–22% of stranded turtles died that were recovered from St. Joseph Bay and Mosquito Lagoon, respectively [13,21]. Cold-stunned turtles recovered in more northerly areas, such as Massachusetts, are typically transported south to Georgia, Florida, or Texas for release [22]. It is challenging to predict large-scale cold-stunning events, making it difficult to adequately plan and budget for federal, state, and non-governmental organizations to mobilize their recovery efforts, especially in years when large numbers of turtles are cold-stunned.

The most common species to cold-stun in the northwest Atlantic is the Kemp’s ridley, followed by loggerheads. Both species use the nearshore waters of the northeastern United States as developmental habitats, including New England, Long Island Sound, and Chesapeake Bay [18,23–26]. Due to thermal constraints, these juvenile turtles must migrate south to warmer waters in fall [27,28]. Juvenile sea turtles become cold-stunned as sea surface temperatures drop to around 10°C, [12,18,29] with death occurring at temperatures ranging from 5.0–6.5°C [29]. The semi-enclosed Cape Cod Bay, Massachusetts, acts as a natural catchment for turtles migrating south, and the bay accounts for most of the cold-stunned turtles in the northeastern U.S. [18]. In Cape Cod Bay, juvenile Kemp’s ridleys (approximately three years old) typically cold-stun in November, while the larger bodied loggerheads can withstand colder water temperatures and typically cold-stun in December [18]. Within the past 40 years, over 4,700 Kemp’s ridleys have stranded within Cape Cod, these cold-stunning events have intensified annually, requiring greater investment in recovery efforts [18]. Prior to 2009, only two years (i.e., 1999 and 2002) had over 100 sea turtles stranded, since 2009, over a hundred sea turtles commonly strand from cold-stunning each year in Cape Cod Bay.

Little is known about what factor(s) drive this increasing number of sea turtle strandings in Cape Cod Bay. A variety of potential factors have been identified to explain this increasing turtle cold-stunning trend, such as changing oceanic and atmospheric conditions, increasing sea surface temperatures, or recovering turtle nesting populations. The objectives of this study were to 1) identify what factors are affecting Kemp’s ridley cold-stunning events in Cape Cod Bay, and 2) predict future rates of cold-stunning based on climate change projections.

## Materials and methods

### Cold-stunned turtle data

Sea turtle cold-stunning data were provided by the Sea Turtle Stranding and Salvage Network (STSSN, https://www.greateratlantic.fisheries.noaa.gov/protected/stranding/disentanglements/turtle/stssn.html, S1 Table), which is coordinated by the National and Oceanic Atmospheric Administration (NOAA). This network is made up of trained stranding responders coordinated locally and by state STSSN coordinators. STSSN has monitored Cape Cod Bay beaches since 1979. With stranding responders monitoring all potential stranding beaches from October-January, we assumed a high probability of locating all stranded turtles for any given year. We used the overall count of found Kemp’s ridley stranded turtles per year from 1982–2016. Stranding data from years prior to 1982 were omitted because of limited availability of sea surface temperature (SST) data.

### Environmental and biological data

**Fig 1:**
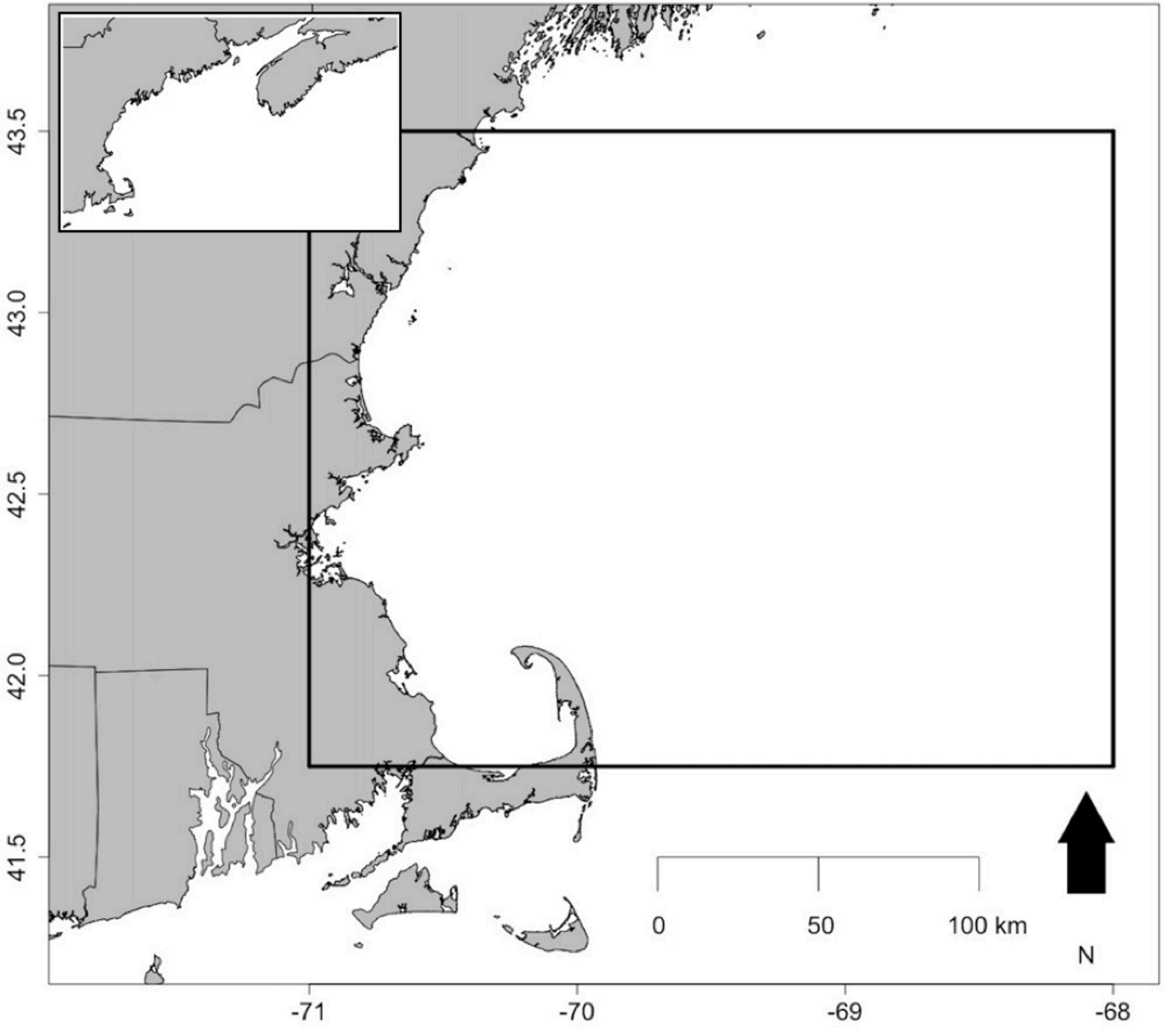
Map of study area that spans across Cape Cod Bay, Gulf of Maine, and a portion of Georges Bank. Sea surface temperature compiled at 25 x 25-degree grids (black boxes) across the area, using the Optimal Interpolation Sea Surface Temperature database from NOAA.

Using the Optimal Interpolation Sea Surface Temperature database (OISST, https://www.ncdc.noaa.gov/oisst) from NOAA, we calculated the average daily SST from 1982–2016 at 25 x 25-degree grids for an area that spans across Cape Cod Bay, Gulf of Maine, and a portion of Georges Bank (Fig 1). This nearly 50,000 km^2^ area encompasses the greater northern area of Cape Cod Bay and Gulf of Maine, where Kemp’s ridleys are likely to occur prior to migrating south in the fall. This area was also chosen to capture the larger scale oceanic thermal conditions that may influence the immigration and emigration of turtles into coastal areas of the northeastern U.S. To examine the relationship between SSTs and cold-stunning events, we derived six aggregate SST statistics at different time scales. These aggregate SST statistics include mean, maximum, minimum, standard deviation of daily mean SSTs, number of days with daily mean SST below 10°C, and number of days with daily mean SST above 20°C. Number of days with daily mean SST below 10°C was chosen because the onset of cold-stun symptoms begins at 10°C [12,18,29]. Number of days with daily mean SST above 20°C was chosen to capture periods of uncharacteristically warm SSTs.

In addition to SST derived statistics, we derived the sum of monthly North Atlantic Oscillation (NAO) indices of each year between June and September. NAO indices, provided from NOAA (https://www.ncdc.noaa.gov/teleconnections/nao/), are linked with pressure, wind, and temperature conditions [30] that may influence turtle recruitment into coastal areas of the northeastern U.S. We chose the months of June through September to represent the period of summer recruitment by turtles into coastal areas (http://www.seaturtlesightings.org/monthmap.html). In addition, the average annual monthly NAO indices were lagged by two years, which infers the latitudinal position of the Gulf Stream for a given year [31]. The annual average monthly Atlantic Multidecadal Oscillation (AMO) indices, unsmoothed (https://www.esrl.noaa.gov/psd/data/correlation/amon.us.data), were derived on an annual basis and also lagged by 1, 2, and 3 years. AMO has been suggested to influence ocean circulation patterns [32], which may affect emigration of juvenile Kemp’s ridleys from the Gulf of Mexico into the Gulf Stream or from the greater Atlantic into Cape Cod. Although the majority of the Kemp’s ridley population comes from the Rancho Nuevo area of Tamaulipas, Mexico [33], we used the annual number of hatchlings released from the Tamaulipas index beaches (Rancho Nuevo, Playa Dos-Barra Del Tordo, Barra Ostionales-Tepehuajes) to examine the role of hatchling numbers on cold-stunning events (hatchling data pre-2015 provided by NMFS & USFWS [34], hatchling data from 2015 and after provided by personal communication Peña, S2 Table). Nesting data were lagged and averaged by 2, 3, and 4 years because Kemp’s ridleys found in Cape Cod are believed to be largely clustered across these years of age (personal communication Avens). All data were examined for outliers and collinearity. All statistical analyses were carried out using the software R [35] (version 3.4.2).

### Climate time windows

We used a sliding window approach [36,37] to determine the optimal climate time window for each of the six aggregate SST statistics (mean, maximum, minimum, standard deviation of daily mean SST, and number of days with daily mean SST below 10°C, and number of days with daily mean SST above 20°C). We also used the climwin package [36] to test multiple hypotheses about the relationship between the climate variables and the biological response. Using annual cold-stunning data as the biological response, the slidingwin function was used to test for and produce, via Akaike information criterion, the best possible climate time window for each aggregate SST statistic. Since the climate time windows were provided at a daily level (ordinal days), we collapsed time windows into half months. If the optimal day was between the 1^st^ and 14^th^ day of a month, it was considered as the early half of the month and if the optimal day was after the 14^th^ day of a month, it was considered as the late half of the month.

### Random forest

We used random forest models from the randomForest package [38] to identify the most important variables in relation to annual Kemp’s ridley cold-stunning counts. Random forest models have relaxed assumptions (i.e., collinearity) and high explanatory power [39,40]. These random forest models, a type of machine learning algorithm, generate and fit hundreds to thousands of decision trees to a data set. However, each decision tree is a bootstrap sample of the total data set and each decision tree searches through a random subset of the predictor variables at each node (decision) location. The best predictor variable for a specific node is chosen and the decision tree proceeds to the next node where a new random subset of predictor variables are evaluated. This is repeated for all subsequent nodes. The random forest model then assesses each variables’ importance by evaluating the decreasing accuracy of trees when each variable is removed, this value is called the mean decrease in accuracy. We first ran the random forest model on all 11 explanatory variables to choose the top two variables that best explained cold-stunning counts. We then eliminated the collinear aggregate SST variables that had the lowest mean decrease in accuracy and subsequently ran the model again with the top non-collinear SST variables (minimum and standard deviation of daily mean SSTs, number of days with daily mean SST below 10°C, and number of days with daily mean SST above 20°C). Initial exploration indicated ~ 500 trees were sufficiently stable for random forest models; however, the number of trees was set to 2,000 for all random forest models to ensure optimal performance was reached.

### Bayesian count model and validation

With a relatively small number of observations (n = 35 count years between 1982–2016), we decided to only use the two most important variables in our count model. Using the two most important variables as identified by our second random forest model, minimum of daily mean SSTs (2nd half of October thru the 1st half of November) and number of hatchlings (lagged by two, three, four years and averaged), we modeled annual Cape Cod Bay Kemp’s ridley cold-stunning counts using a negative binomial distribution with approximate Bayesian inference models using Integrated Nested Laplace Approximations in the INLA package [41,42]. A Bayesian framework provides a posterior distribution for each parameter, and thus we can infer the unknown parameter is 95% likely to fall within a range of values around each posterior distribution as defined by the 0.025 and 0.975 quantiles. INLA, an alternative to Markov Chain Monte Carlo method, provides an efficient tool to obtain posterior distributions using numerical approximations [43].

Further, we modified variance estimates around each parameter using informative prior distributions to include measurement error of our selected covariates. Ignoring measurement error may severely bias parameter estimates and credible intervals, resulting in misinterpreting real covariate signals [44]. When expert and prior knowledge exists about the uncertainty of the explanatory variables, it is possible to incorporate measurement error into the model via Bayesian analyses [44,45]. We applied a heteroscedastic error structure (i.e., error changes from observation to observation) to the minimum of daily mean SSTs, which was derived from the OISST platform (mean ± SD = 0.19± 0.02°C). We applied a homoscedastic error structure (i.e., error remains a constant value across observations) to the number of hatchlings parameter. Since observation error was not reported for hatchlings released, we derived our informative prior for this parameter by calculating the standard deviation of the last 10 years of hatchlings released and then scaled each observation by this standard deviation. The counts of hatchlings released from the last 10 years were used because the trend appears to asymptote during this period; thus, the variation from observation to observation in these last 10 years may be indicative of some measurement error across the entire trend.

Multiple structures, including first-order autoregressive and first and second-order random walks, were applied and assessed, following Zuur et al. [46], to address potential issues with temporal autocorrelation. However, all autocorrelation structures led the model to over fit the data, and we decided to exclude these structures. Further, we decided not to include year as a covariate due to high collinearity (assessed via variance inflation factors) with the two parameters of minimum of daily mean SSTs and number of hatchlings.

We performed backwards step-wise model selection using Deviance Information Criterion (DIC, [47]), and autocorrelation was assessed using the acf function from the stats package [35]. The final model was examined for overdispersion and homogeneity by plotting the residuals against fitted values, and for potential patterns in residuals by plotting residuals versus each covariate in the model and each covariate not in the model. In addition, we evaluated the models performance by assessing the fitted and observed values using the full dataset.

### Prediction

The final model of annual Kemp’s ridley cold-stunning counts as a function of minimum of daily mean SSTs (2^nd^ half of October thru the 1^st^ half of November) was then used to predict the potential future trend of Cape Cod Bay annual Kemp’s ridley cold-stunning counts with warming sea surface temperatures. Predicted SSTs were derived specifically for our constructed study area using the observed minimum of daily mean SSTs rather than from global climate models that have multiple climatic scenarios. To generate estimates for potential future SSTs, we first calculated the slope of the observed minimum of daily mean SSTs and an intercept from the mean minimum of daily mean SSTs for the last 15 years. Using the calculated slope and intercept, we generated predicted temperatures for 15 years in advance of our study period. We set the measurement error for all 15 predicted temperature values to 0.5, generating additional uncertainty around these values.

Using both our future cold-stun predictions, as related to SST warming via climate change, and population estimates derived from Gallaway et al. [48], we roughly estimated the potential future population level effect of cold-stunning. Gallaway et al. [48] reported the 2012 estimated Kemp’s ridley female population at age two, three, and four were 32,060, 23,057, and 22,918, respectively. Female sex ratios were reported at 0.65 and 0.74 for in situ and protected nests, respectively. Using the averaged female population age estimate at age two, three, and four, and the averaged female sex ratios, as reported by Gallaway et al. [48], we estimated the total number of turtles at age two, three, and four and then compared this estimate with our mean 2031 cold-stun prediction count. This assumes the population estimate of 2012 remains the same until 2031.

## Results

**Fig 2:**
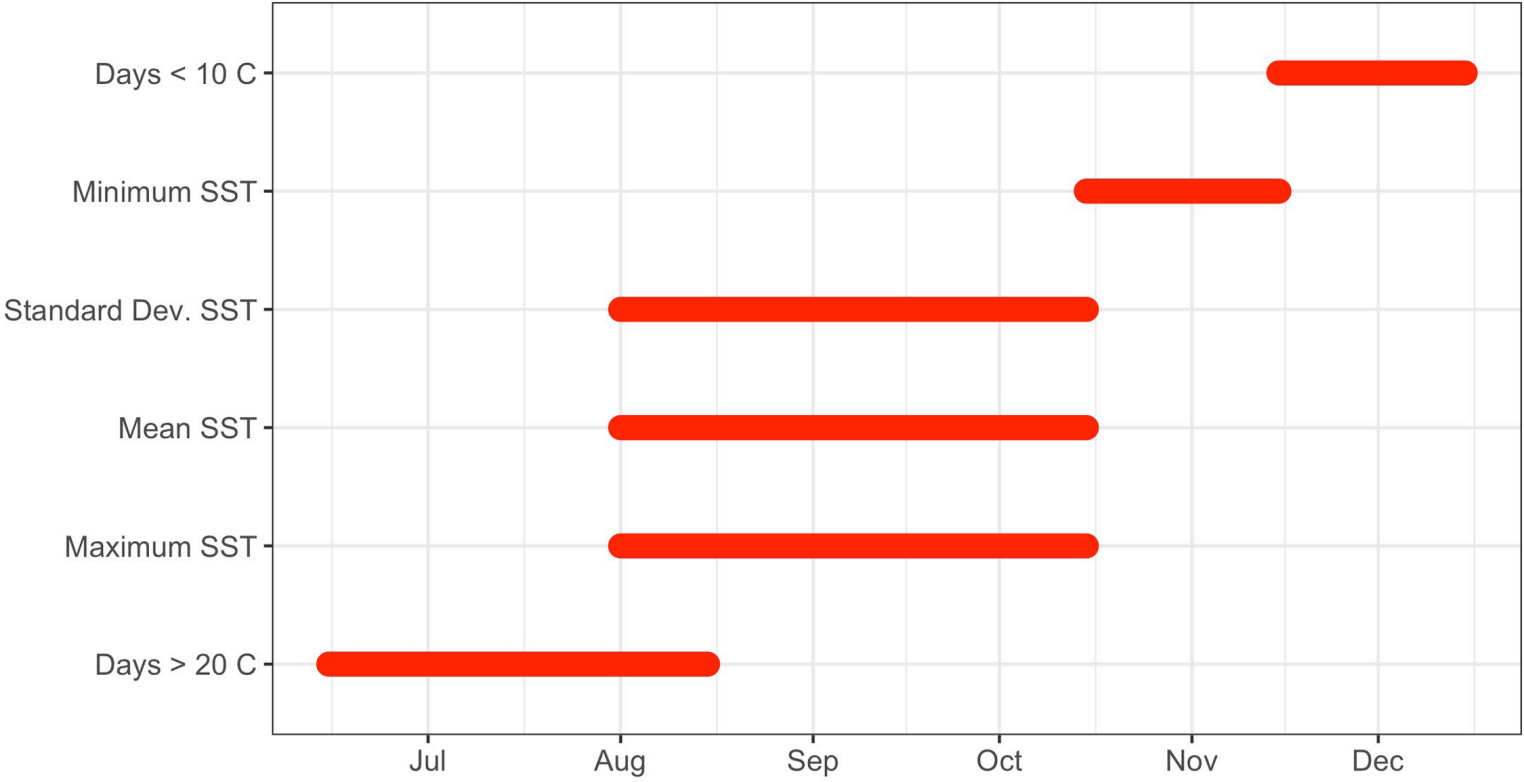
Optimal climate time windows for selected variables. As identified by the climwin package [35] for each aggregate SST statistic, including: mean, maximum, minimum, and standard deviation of the daily mean SSTs, number of days with daily SST below 10°C, and number of days with daily SST above 20°C.

With the wide variation in SSTs both seasonally and between years, we first used the sliding window approach to determine the optimal time window for examining the relationship between SST and cold-stunning events. The optimal climate time windows differed for the six aggregate SST statistics (Fig 2). The earliest time window occurred from late June thru early August for number of days with daily mean SST > 20°C. The optimal time window for three of the SST statistics (mean, maximum, and standard deviation of the daily mean SSTs) occurred from early August thru the first half of October. The third time window occurred from late October thru early November for the minimum of the daily mean SSTs, and from late November thru early December for number of days with daily mean SST < 10°C.

**Fig 3:**
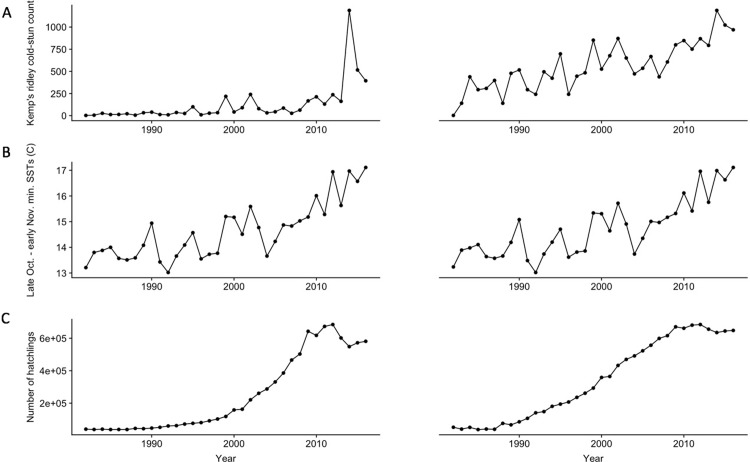
Time series plots. Raw (left) and logged (right) time series (1982–2016) of A) annual Kemp’s ridley cold-stun counts within Cape Cod Bay, B) minimum of the daily mean SST across late October thru early November within the study area, and C) number of hatchlings (lagged by two, three, four years and averaged).

Minimum of daily mean SSTs and number of hatchlings were the two most important variables associated with annual Kemp’s ridley cold-stunning counts (Fig 3) identified by the random forest models. Consequently, we removed the two SST variables mean and maximum of daily mean SSTs from the second random forest model due to collinearity.

**Fig 4:**
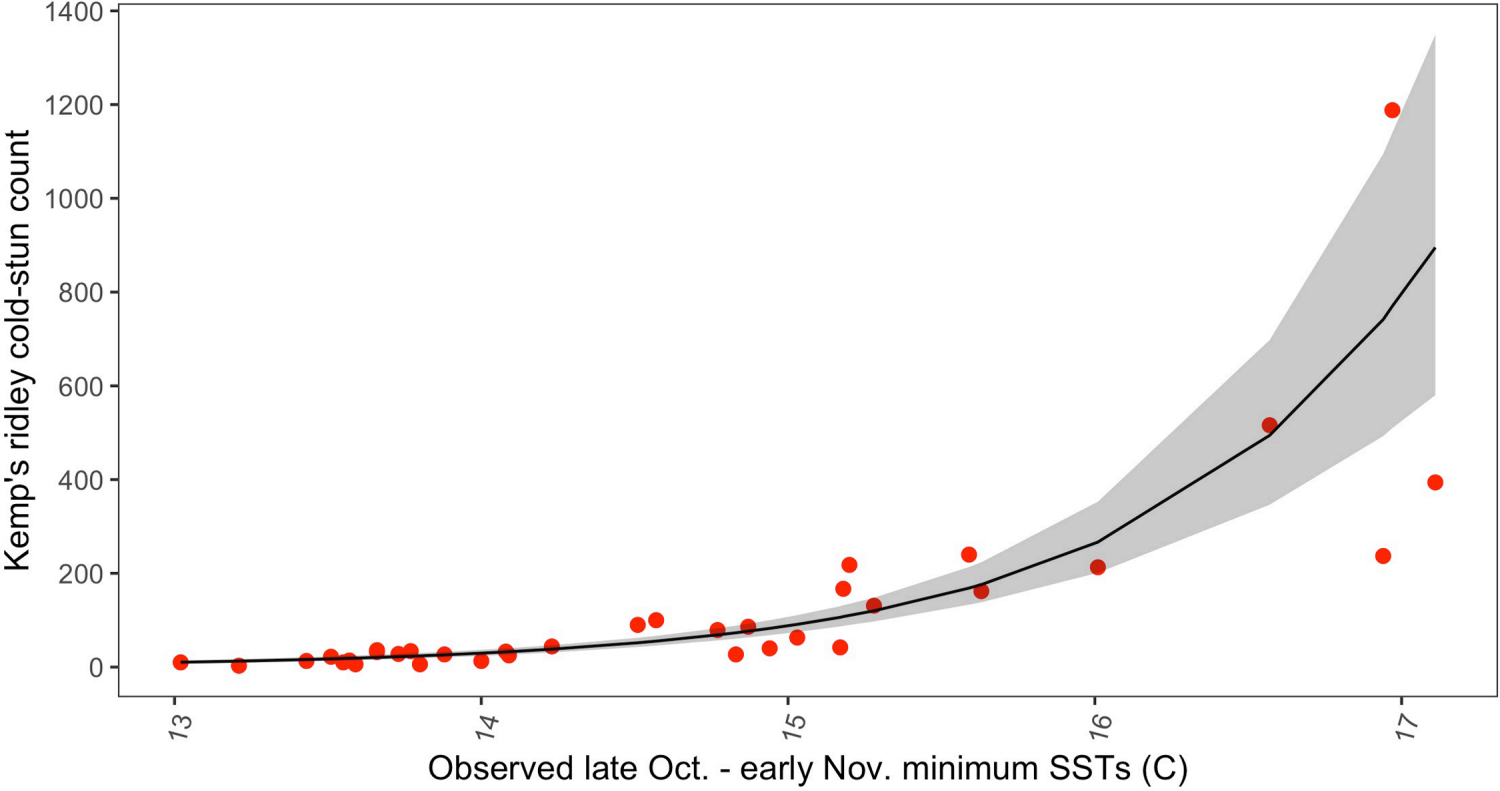
Kemp’s ridley cold-stun count versus minimum of the daily mean SST from late October thru early November. Included are posterior mean fitted values and 95% credible intervals.

Both minimum of daily mean SSTs and number of hatchlings along with their respective measurement error structures were included in the Bayesian count model (DIC 341.22). Using number of hatchlings alone as a covariate within the model produced a much higher DIC value of 380.55; however, using minimum of daily mean SSTs alone as a covariate produced a DIC value of 339.41. We decided to drop number of hatchlings from our final model based on the slightly lower DIC value and because number of hatchlings was found not to be important in original model (number of hatchlings, posterior mean = -0.02; 95% Credible Intervals (CI) = - 0.3, 0.25]). Thus, using a negative binomial distribution, minimum of daily mean SSTs during late October thru early November was the variable that best explained annual cold-stunning counts. Annual Kemp’s ridley cold-stunning events were more likely to be higher when the corresponding late fall SSTs were warmer (Fig 4, minimum of daily mean SSTs, posterior mean = 1.23; 95% Credible Intervals (CI) = 1.04, 1.41]).

Negative temporal autocorrelation at a lag of two was still present among the residuals, but it was minor and largely accounted for by using temperature as a covariate. Further, no obvious trend in the residuals existed, indicating little effect of temporal autocorrelation. The calculated overdispersion statistic was 1.02, indicating no overdispersion issues and the negative binomial was an adequate probability distribution. Common with count models, deviations from the expected increased with larger expected values, which occurred within this dataset in the later years. The observed values from the full dataset were heavily outside the final model’s 95% CI; yet, the fitted trend appeared to closely match the trend in the observed data (Fig 4).

**Fig 5:**
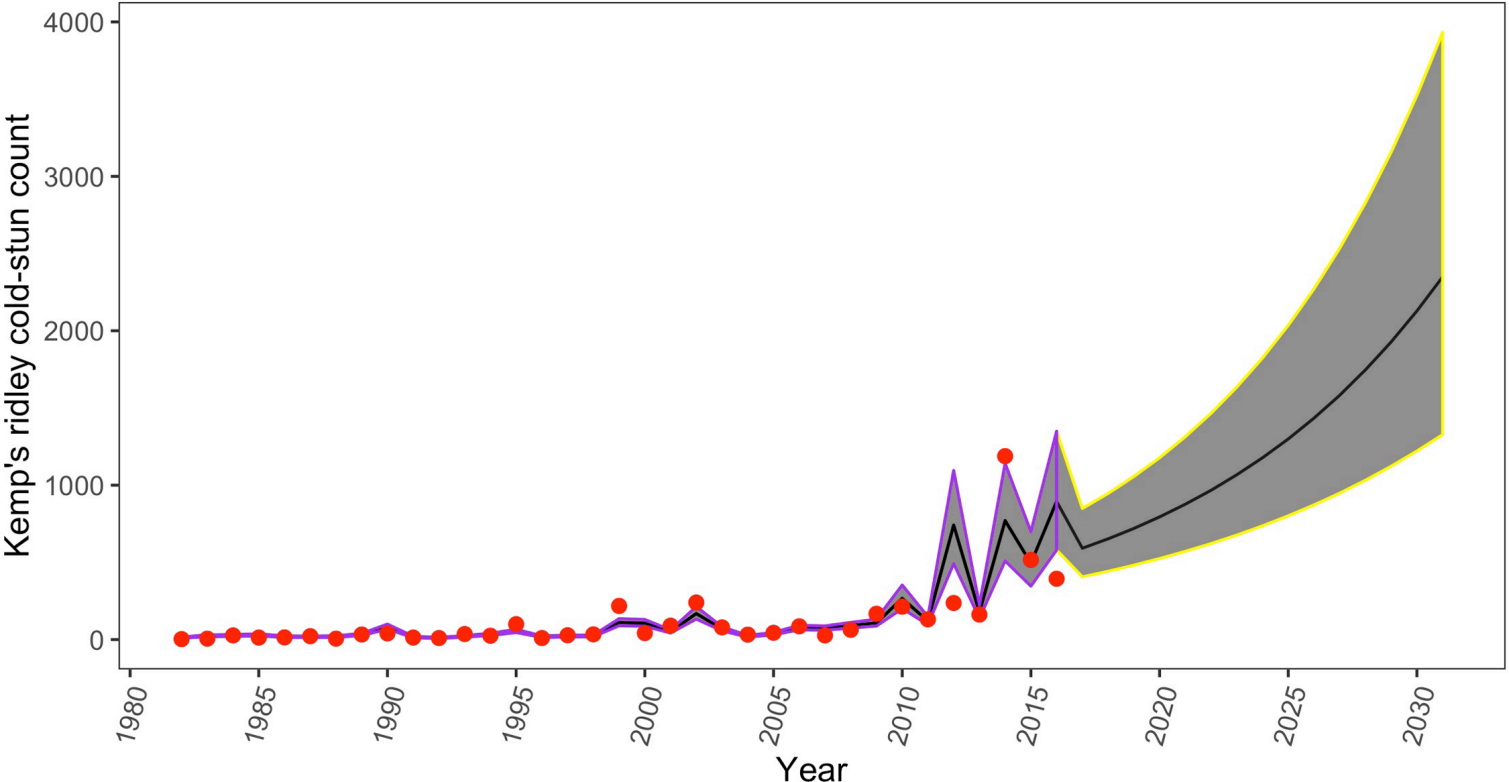
Observed and predicted Kemp’s ridley cold-stun count based on predicted future minimum of the daily mean SST (late October thru early November) within the study area.

Assuming SSTs within the Northwest Atlantic will continue to increase in the future [47], we generated predicted temperatures (mean ± SD = 17.36 ± 0.40°C) for 15 years in advance of our study period using the slope and intercept of observed minimum of daily mean SSTs in the past 15 years. Since these predicted temperatures may vary, we incorporated a measurement error of 0.5 to all temperatures to incorporate more realistic uncertainty. Using these predicted temperatures and their associated measurement errors, we forecasted annual Kemp’s ridley cold-stunning counts 15 years into the future. By 2031, the posterior mean predicted Kemp’s ridleys cold-stunning count was 2,349 (95% CI = 1,328, 3,933) (Fig 5).

If the Kemp’s ridleys age structure as reported by Gallaway et al. [48] remains the same for the next 15 years, we estimate that approximately 1.8% of the juveniles (age classes two, three, and four) may cold-stun by 2031.

## Discussion

Our study indicates that warming SST in the Gulf of Maine are associated with the increasing numbers of Kemp’s ridley cold-stunned in Cape Cod Bay each year. The minimum of daily mean SSTs, alone, measured between late October thru early November, best explained the magnitude of annual Kemp’s ridley cold-stunning events in Cape Cod Bay. However, maximum and mean of daily mean SSTs, both measured between August and early October, were collinear with minimum of daily mean SSTs. Thus, while warmer SSTs in late fall are indicative of higher annual cold-stunning counts, so are warmer SSTs in late summer and early fall. While our Bayesian count model found SST to be the most important variable in explaining the number of cold-stunned Kemp’s ridleys, the model would be improved with a greater understanding of the small and large scale oceanic processes at work, such as eddies, currents, and thermoclines, which all operate on multiple spatial and temporal scales. However, our single covariate likely acts as a proxy for these processes, and our model does appear to explain the observed Kemp’s ridleys cold-stunning trend. Surprisingly, the covariate number of hatchlings was not considered important in our full candidate count model, so we dropped this variable from the final model. Although the Kemp’s ridley nesting population has increased over the years of our analyses, our results suggest the number of hatchlings released is not linked with the magnitude of cold-stunning events in Cape Cod Bay. Potentially, this statistical relationship between strandings and hatchlings was dampened due to variable hatchling survival (based on surface circulation patterns near nesting beaches) and due to the variable probability of turtles moving from the western Gulf of Mexico nesting beaches into the Atlantic [49]. However, the hatchling indices do provide our best insight into the potential connection between population growth and cold stunning events.

Over the last decade, SSTs are warming 99% faster than the global ocean within the Gulf of Maine [50]. These warmer SSTs may be allowing Kemp’s ridley to expand their northerly distribution along the northeast Atlantic continental shelf, as reported for many fish species [51]. Although numbers of Kemp’s ridley cold-stun strandings increased in both Cape Cod Bay and Long Island Sound since the 1970s, the magnitude of sea turtles cold-stunned have increased dramatically within Cape Cod Bay in comparison to Long Island Sound. This supports the hypothesis that the Kemp’s ridley northerly neritic developmental grounds may have shifted more northward along the Atlantic coast, potentially in response to warming SSTs in the Gulf of Maine. Although Carr [52,53] suggested that neonate and juvenile sea turtles disperse passively with wind and currents, Putman et al. [54], Mansfield et al. [55], and Putman and Mansfield [56] demonstrated juvenile turtles are highly capable of active dispersal. Further, Mansfield et al. [55] tracked neonate loggerheads with satellite telemetry and showed turtles may select for sea surface habitats based on thermal constraints. If these warmer thermal habitats are driving turtles to recruit to more northerly neritic developmental grounds, Cape Cod Bay may act as a natural catchment during the southerly migration in colder months. As suggested by Briscoe et al. [57], if the warmer Gulf of Maine temperatures are acting as an ecological bridge that promotes higher levels of recruitment of organisms into nearshore waters from the Gulf Stream, numbers of cold-stunned Kemp’s ridley turtles may well continue to increase over time as suggested by our mean prediction of 2,349 (95% CI = 1,328, 3,933) cold-stunned by 2031.

While we were unable to explain all outlier cold-stun years (1999, 2002, and 2014), we suggest Hurricane Arthur may have contributed to the high cold-stun count in 2014 (n = 1,188). Hurricane Arthur (1–5 July, 2014, https://www.nhc.noaa.gov/data/tcr/AL012014_Arthur.pdf), a category 2 hurricane (on Saffir-Simpson Hurricane Wind Scale) was an unusually early and severe hurricane to hit the northeast U.S. This storm may have 1) warmed waters, promoting sea turtle immigration into nearshore areas, or 2) generated enough wind and current to force sea turtles into nearshore waters [58]. Since we were unable to assess this with our methods, we suggest future studies to consider anomalous hurricanes as potential predictors for atypically large cold-stunning events.

When evaluating the importance of cold-stunning recovery and rehabilitation efforts in the northeast U.S., it is important to consider the proportion of the Kemp’s ridley population affected, and whether juvenile Kemp’s ridleys in the Atlantic return to the Gulf of Mexico to reproduce. At the population level, cold-stunning may be affecting only a small fraction of the overall Kemp’s ridley population. Assuming the Kemp’s ridley age structure proportions reported by Gallaway et al. [48] remain the same over time, we estimate that less than 2% of the juveniles within age classes two, three, and four may be cold-stunned in 2031. If there were no cold-stun recovery or rehabilitation efforts in 2031, cold-stunning deaths, estimated at 2,349 turtles, would only be a small fraction of mortality for the projected overall Kemp’s ridley population. Further, juvenile survivorship is often not considered as critical for population growth in comparison to the survivorship of larger sub-adult and adult turtles [59–61]. However, depending on the future and variable Kemp’s ridley population demographics, cold stunning events may eventually account for a larger proportion of the population if more turtles are recruiting northward. We also do not know to what extent juvenile Kemp’s ridleys on the Atlantic coast return to the Gulf of Mexico to reproduce, but it has been suggested that turtles found on the Atlantic coast may have the navigational abilities to migrate back to the Gulf of Mexico [62–64]. Despite the potentially small effect on the overall population, we believe that it is important to continue recovery and rehabilitation efforts for juvenile cold-stunned Kemp’s ridleys in the northeast U.S. to bolster population resiliency. This increased resiliency is important considering the slowing trend of nesting Kemp’s ridley females and continuing anthropogenic threats to turtles in the Gulf of Mexico [33,48,65–69]. Thus, we recommend that all conservation efforts, including the rehabilitation of cold-stunned Kemp’s ridleys, be continued for this critically endangered species.

## Conclusion

Cold-stunning of Kemp’s ridleys within Cape Cod Bay has continued to increase over the past 40 years. Our model indicated that years with warmer SSTs in the Gulf of Maine in late summer thru late fall produce higher numbers of cold-stun turtles on an annual basis. This is particularly alarming, considering the Gulf of Maine is predicted to continue to warm at a rapid rate in coming decades [50]. Surprisingly, hatchlings released, a proxy for population abundance, was not identified as important by our Bayesian count model. Our predictions follow the observed trend and predict there may be as many as 2,349 Kemp’s ridley turtles cold-stunned annually in Cape Cod Bay by 2031. Although cold-stunning likely only affects a small proportion of the overall population currently, we argue for the continuation of recovery and rehabilitation efforts to help maintain population resiliency of this critically endangered species. As we continue to observe warming SSTs in the northeast U.S. driven by climate change, managers need to be prepared for increasing numbers of Kemp’s ridley cold-stun strandings to occur. Future studies should 1) determine when Kemp’s ridleys typically immigrate into and emigrate out of coastal waters of the northeastern U.S., and 2) if juvenile Kemp’s ridley turtles migrate back into the Gulf of Mexico to breed as adults.

## Supporting information

S1 Table Annual Kemp’s ridley cold-stun stranding count (1982–2016) from Cape Cod, Massachusetts, USA. Sea turtle cold-stunning data provided by the Sea Turtle Stranding and Salvage Network.
(CSV)

S2 Table Annual number of hatchlings released (1966–2018) from the Tamaulipas index beaches (Rancho Nuevo, Playa Dos-Barra Del Tordo, Barra Ostionales-Tepehuajes). Hatchling data pre-2015 provided by NMFS & USFWS 2015, hatchling data from 2015 and after provided by personal communication Peña.
(CSV)
